# A Literature Review of Zika Virus

**DOI:** 10.3201/eid2207.151990

**Published:** 2016-07

**Authors:** Anna R. Plourde, Evan M. Bloch

**Affiliations:** University of California, San Francisco, California, USA (A.R. Plourde);; The Johns Hopkins University, Baltimore, Maryland, USA (E.M. Bloch); Blood Systems Research Institute, San Francisco (E.M. Bloch)

**Keywords:** Zika virus, arbovirus, flavivirus, viruses, emerging infectious diseases, zoonoses, microcephaly, craniofacial abnormalities, mosquitoes, Aedes, review literature as topic

## Abstract

Zika virus is a mosquitoborne flavivirus that is the focus of an ongoing pandemic and public health emergency. Previously limited to sporadic cases in Africa and Asia, the emergence of Zika virus in Brazil in 2015 heralded rapid spread throughout the Americas. Although most Zika virus infections are characterized by subclinical or mild influenza-like illness, severe manifestations have been described, including Guillain-Barre syndrome in adults and microcephaly in babies born to infected mothers. Neither an effective treatment nor a vaccine is available for Zika virus; therefore, the public health response primarily focuses on preventing infection, particularly in pregnant women. Despite growing knowledge about this virus, questions remain regarding the virus’s vectors and reservoirs, pathogenesis, genetic diversity, and potential synergistic effects of co-infection with other circulating viruses. These questions highlight the need for research to optimize surveillance, patient management, and public health intervention in the current Zika virus epidemic.

## Abstract 2

We summarize what is known about this virus and its global expansion as of mid-February 2016.

Zika virus is a flavivirus that was first isolated in 1947 from a febrile rhesus macaque monkey in the Zika Forest of Uganda and later identified in *Aedes africanus* mosquitoes from the same forest (*1*). In 1954, the first 3 cases of human infection were reported in Nigeria (*2*). Serosurveillance studies in humans suggest that Zika virus is widespread throughout Africa, Asia, and Oceania (Technical Appendix Table 1). However, these studies may overestimate the virus’s true prevalence, given serologic overlap between Zika virus and related flaviviruses, such as dengue virus (DENV) and West Nile virus (WNV) (*3**,**4*).

Historically, symptomatic Zika virus infections were limited to sporadic cases or small clusters of patients (Technical Appendix Table 2). This pattern changed in 2007, when the first major outbreak of Zika virus infection occurred in Yap (Federated States of Micronesia), where ≈73% of the population were infected and symptomatic disease developed in ≈18% of infected persons (*5*). Since then, Zika virus infection has spread rapidly. Outbreaks have occurred in French Polynesia (*6*), Cook Islands (*6*), Easter Island (*7*), New Caledonia (*8*), and, most recently, the Americas (*9*), with sporadic exportations to Europe (Figures 1,2,3; Technical Appendix Table 2).

**Figure 1:**
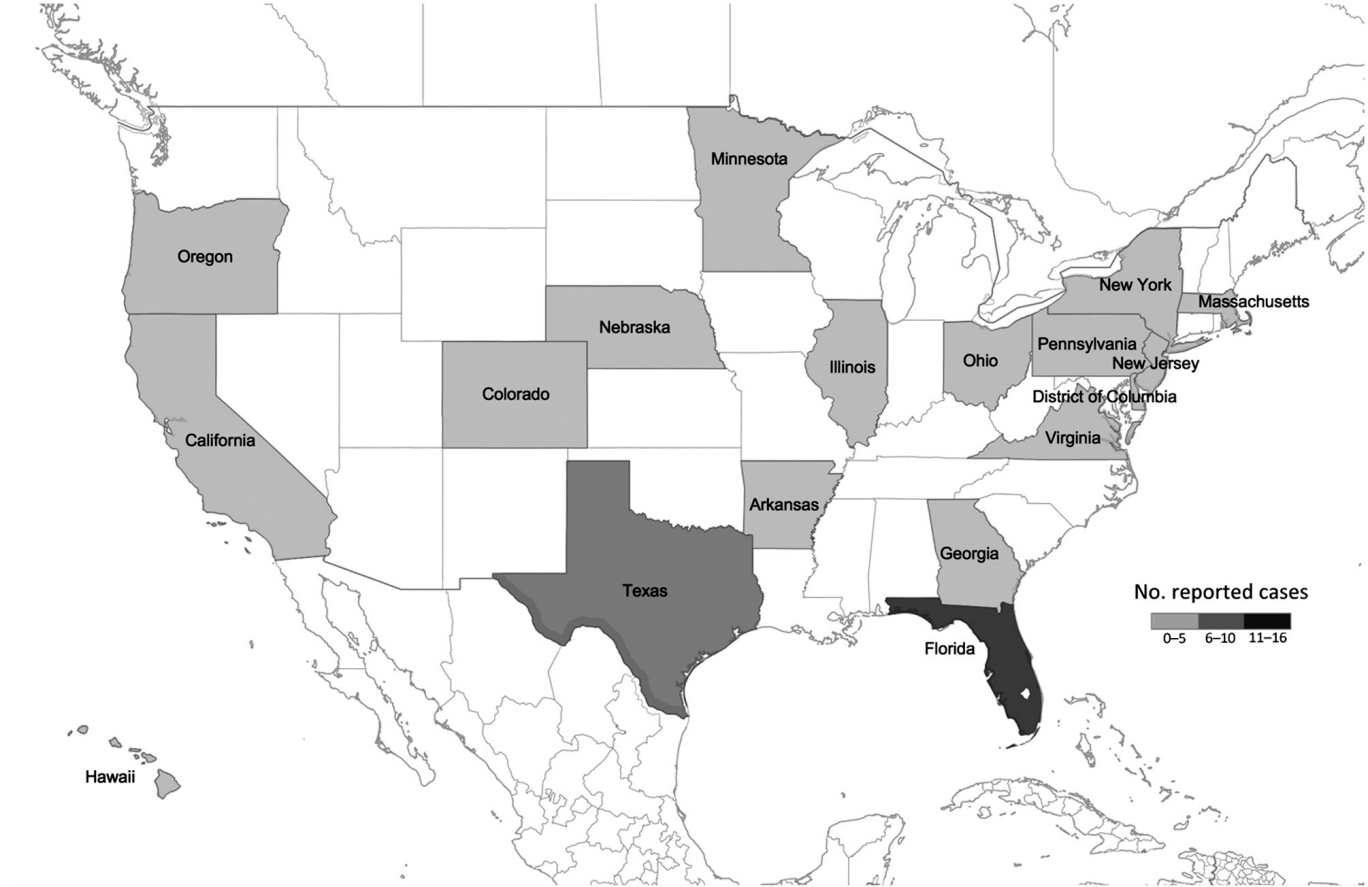
Cases of laboratory-confirmed, imported Zika virus infections in the United States, by state, January 1, 2015–February 10, 2016 (*10*)*.* All cases are imported, with the exception of 2 sexually acquired autochthonous cases (*11**,**12**).*

**Figure 2:**
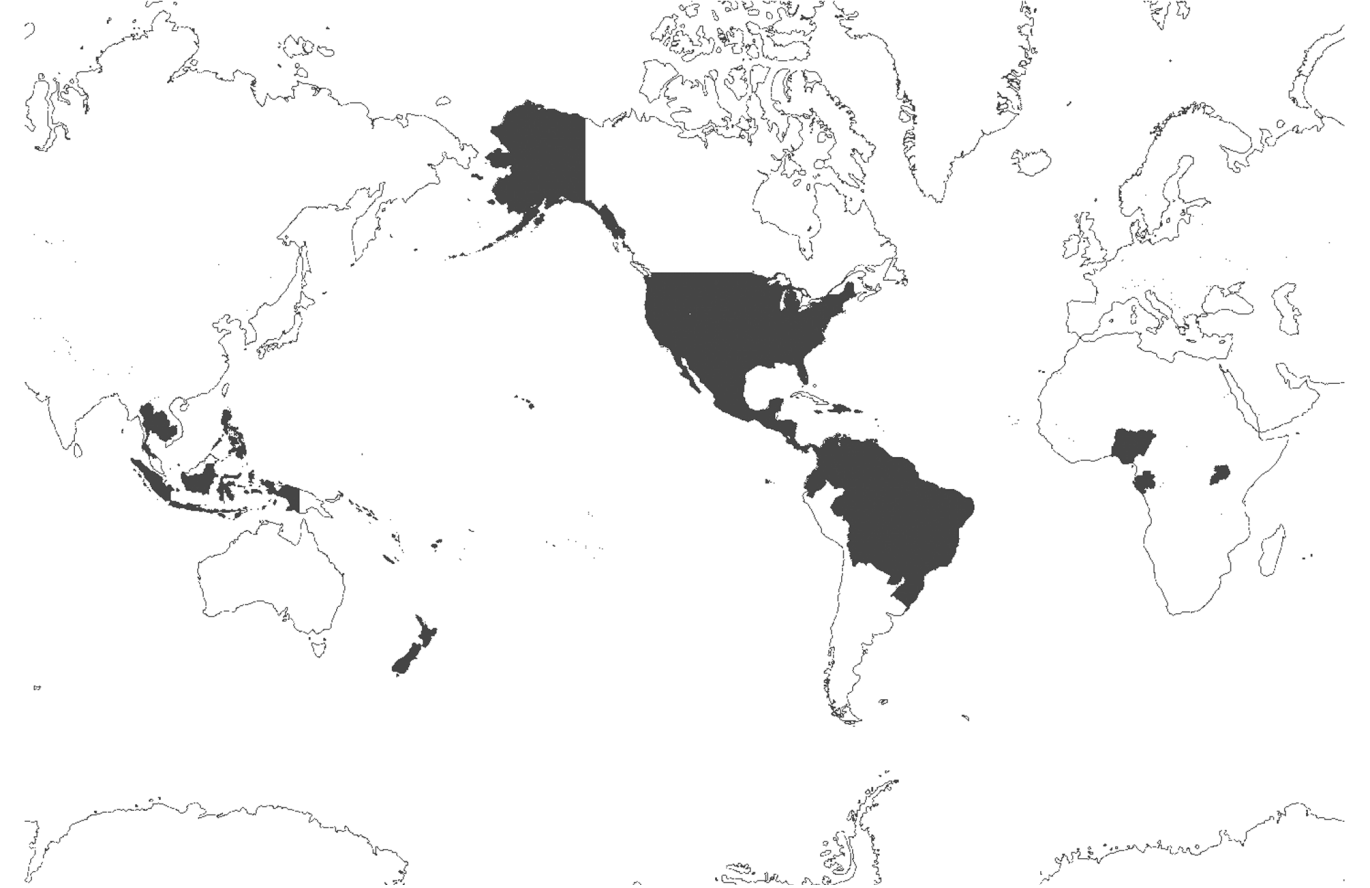
All countries and regions reporting laboratory-confirmed autochthonous Zika virus cases, January 1, 2015–February 10, 2016 (Technical Appendix Table 2). Data represent outbreaks and case reports for all reported autochthonous laboratory-confirmed cases of Zika virus infection, including those reported in the peer-reviewed literature; public health agency Web sites, bulletins, and broadcasts; and media reports for selected dates.

**Figure 3:**
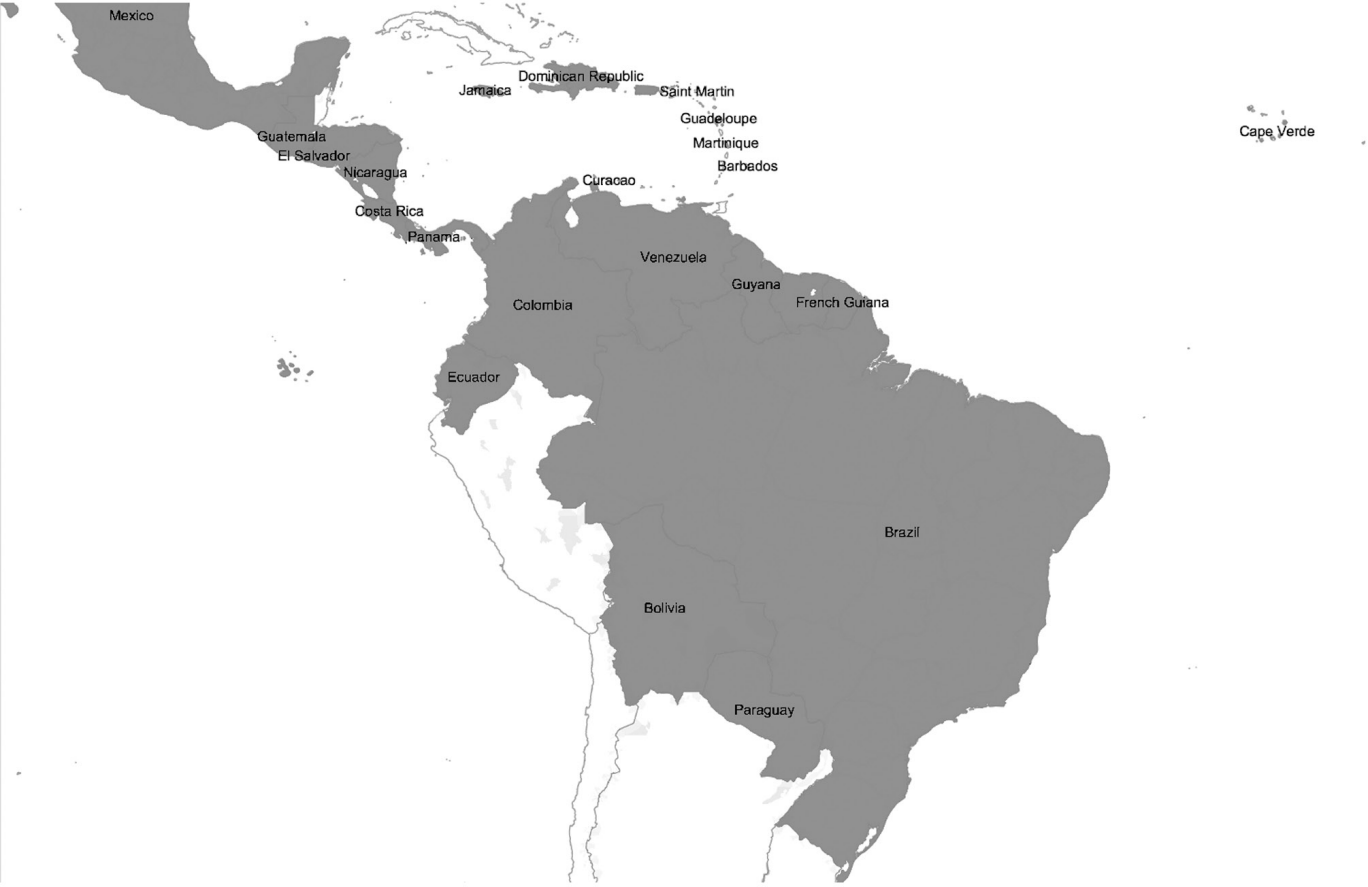
South America, Central America, and Caribbean countries and regions reporting laboratory-confirmed autochthonous Zika virus disease cases during January 1, 2015–February 10, 2016 (Technical Appendix Table 2). Data represent outbreaks and case reports for all reported autochthonous laboratory-confirmed cases of Zika virus infection in these countries and regions during January 1, 1952–February 10, 2016, including those reported in peer-reviewed literature; public health agency Web sites, bulletins, and broadcasts; and media reports.

Zika virus was first reported in May 2015 in continental South America in Brazil, where ≈440,000–1,300,000 persons have subsequently been infected (as of February 16, 2016). Furthermore, 29 other countries in the Americas have reported autochthonous Zika virus transmission, including Puerto Rico and US Virgin Islands (Figure 3; Technical Appendix Table 2) (*13*). Except for 2 sexually acquired cases, Zika virus in the United States, Canada, and Europe has been restricted to travelers from affected areas (Figure 1; Technical Appendix Table 2); a patient who delivered an infant with microcephaly in Hawaii had spent part of her pregnancy in Brazil (*14*).

Given the wealth of new information about Zika virus, we conducted a literature review to summarize the published findings. This review contextualizes the ongoing Zika virus epidemic in the Americas and identifies knowledge gaps that must be addressed to combat Zika virus successfully.

## The Review

### Search Strategy and Selection Criteria

Using the keywords “Zika,” “ZIKV,” “ZIKAV,” and “Zika virus,” we searched Google, PubMed, Web of Science, Scopus, and ProMed Mail. We reviewed all literature published through February 16, 2016, including peer-reviewed journal articles, infectious disease reporting system broadcasts, and public health agency information (e.g., US Centers for Disease Control and Prevention [CDC] and European Centre for Disease Prevention and Control [ECDC]). To ensure the capture of all information, we cross-referenced the bibliographies of reviewed articles. The search included English-language and foreign-language articles, which were computer translated.

### Virology and Pathogenesis

Zika virus is a positive-sense single-stranded RNA virus in the family *Flaviviridae*, which includes several other mosquitoborne viruses of clinical importance (e.g., DENV, WNV, and yellow fever virus [YFV]) (*15*). Its closest relative is Spondweni virus, the only other member of its clade (*15**,**16*). The Zika virus genome contains 10,794 nt encoding 3,419 aa (*16*). Like other flaviviruses, Zika virus is composed of 2 noncoding regions (5′ and 3′) that flank an open reading frame (*16*), which encodes a polyprotein cleaved into the capsid, precursor of membrane, envelope, and 7 nonstructural proteins (*16*).

Phylogenetic analysis shows that Zika virus can be classified into distinct African and Asian lineages; both emerged from East Africa during the late 1800s or early 1900s (*17*). The Asian lineage originated during the virus’s migration from Africa to Southeast Asia, where it was first detected in Malaysia. From there, Zika virus spread to the Pacific Islands, separately to Yap and French Polynesia, and then to New Caledonia, Cook Islands, Easter Island, and the Americas (*17*).

A study of Zika virus’s molecular evolution, based on viral strains collected from 4 countries in West Africa during 1947–2007, identified several sites within the Zika viral genome that were under strong negative selection pressure. This finding suggests frequent purging of deleterious polymorphisms in functionally important genes and the possibility of recombination, which occurs rarely among flaviviruses (*18*). The implications of this finding require further evaluation with respect to viral spread, zoonotic maintenance, and epidemiologic potential.

After mosquito inoculation of a human host, cellular entry likely resembles that of other flaviviruses, whereby the virus enters skin cells through cellular receptors, enabling migration to the lymph nodes and bloodstream. Few studies have investigated the pathogenesis of Zika virus infection. One study showed that human skin fibroblasts, keratinocytes, and immature dendritic cells allow entry of Zika virus (*19*). Several entry and adhesion factors (e.g., AXL receptor tyrosine kinase) facilitate infection, and cellular autophagy, needed for flaviviral replication, enhances Zika virus replication in skin fibroblasts (*19*). After cellular entry, flaviviruses typically replicate within endoplasmic reticulum-derived vesicles. However, Zika virus antigens were found exclusively in the nuclei of infected cells; this finding suggests a location for replication that differs from that of other flaviviruses and merits further investigation (*20*).

### Transmission

Zika virus, like other flaviviruses, is transmitted by mosquitoes, primarily of the *Aedes* (*Stegomyia*) genus. Several *Aedes* spp. have been implicated, including *Ae. aegypti*, *Ae. africanus*, *Ae. hensilli*, *and Ae. albopictus* (*1**,**21**–**23*). The *Ae. aegypti* mosquito appears to be the major vector in Asia (*24*) and was the suspected primary vector for the French Polynesia outbreak (*25*). Zika virus has been detected in wild-caught *Ae. aegypti* mosquitoes, which laboratory experiments have shown to be capable of transmitting Zika virus (*26**,**27*). *Ae. hensilli* mosquitoes were implicated in the Yap outbreak, yet Zika virus has never been isolated from these mosquitoes (*28**,**29*). In Africa, the predominant *Aedes* species vector has not been definitively identified, although viral isolation studies suggest that *Ae. albopticus* was the likely vector in a 2007 Zika virus outbreak in Gabon (*23*).

*Aedes* mosquitoes are widely distributed globally, and native habitats of most species are warm tropical and subtropical regions (*29**–**31*). Some species show a limited distribution (e.g., *Ae. luteocephalus* in Africa and *Ae. hensilli* in the Pacific Islands); others have a broad geographic span (e.g., *Ae. aegypti* and *Ae. albopictus*) (*29**–**31*). *Ae. albopictus* does not yet appear to be a major vector of Zika virus. However, its role in the 2007 Gabon outbreak, its wide distribution throughout the United States, and Zika virus’s lack of restriction to a specific *Aedes* sp. indicate that this species could serve as a vector in the United States (*9*).

Mosquito acquisition of the virus likely occurs during a blood meal; after uptake, the virus replicates and is transmitted to a reservoir animal at the next blood meal (*32*). Isolation of the virus or of anti-Zika virus antibodies from various nonhuman primates and other wild and domestic animals suggests multiple animal reservoirs (*33*). One study examined the kinetics of Zika virus infectivity in *Ae. aegypti* mosquitoes by using blood-feeding membranes (*27*); viral content was high on the day of feeding (inoculation), decreased to undetectable levels through day 10, increased by day 15, and remained high on days 20–60. These findings suggest an incubation period in mosquitoes of ≈10 days.

Other nonvector modes of Zika virus transmission include congenital (*34*), perinatal (*35*), and sexual (*11**,**36*). Possible transmission by blood transfusion (*37**,**38*), animal bite (*39*), and laboratory exposure (*40*; Technical Appendix reference *41*) has been described; however, confounding by contemporaneous vectorborne transmission in these instances cannot be excluded. For example, the patient who became infected with Zika virus after a monkey bite had concomitant exposure to mosquitoes, a more plausible route of acquisition (*39*). Similarly, 1 of 2 patients with potentially laboratory-acquired infection (*40*; Technical Appendix reference *41*) reported recent exposure to mosquitoes (*40*); no definitive mechanism for transmission was described for either patient.

Intrauterine transmission is supported by the finding of Zika virus RNA by reverse transcription PCR (RT-PCR) in amniotic fluid of 2 mothers with symptoms of Zika virus infection during pregnancy; both delivered babies with microcephaly (*34*). Zika virus RNA has also been identified in tissue of fetuses from women infected during pregnancy and in brains of 2 live-born infants with microcephaly who died <20 hours after birth (Technical Appendix references *42–45*). Probable intrapartum transmission has also been described: 2 newborns were found to be viremic with Zika virus <4 days after being born to infected mothers (*35*). Viral RNA, but not culturable virus, has been detected in breast milk (*35*), but transmission by breast-feeding has not been reported.

Two cases of possible transfusion-transmitted Zika virus were reported in Brazil (*38*). Furthermore, during the French Polynesia outbreak, a study found that 42 (2.8%) of 1,505 asymptomatic blood donors were positive for Zika virus by RT-PCR; 11 donors described a Zika fever-like syndrome 3–10 days after donation (*37*).

### Clinical Manifestations

In humans, the incubation period from mosquito bite to symptom onset is ≈3–12 days. Infection is likely asymptomatic in ≈80% of cases (*5**,**32*). All ages are susceptible (4 days–76 years), with a slight preponderance of cases in females (Technical Appendix Table 3). When symptoms occur, they are typically mild, self-limiting, and nonspecific (Technical Appendix Table 3); similarity to other arbovirus infections (e.g., DENV and chikungunya virus [CHIKV]) may confound the diagnosis (Technical Appendix reference *46*). Commonly reported symptoms include rash, fever, arthralgia, myalgia, fatigue, headache, and conjunctivitis (Technical Appendix Table 3). Rash, a prominent feature, is maculopapular and pruritic in most cases; it begins proximally and spreads to the extremities with spontaneous resolution within 1–4 days of onset (*40*). Fever is typically low grade (37.4**°**C –38.0**°**C) (*8**,**36**,**40*). Symptoms resolve within 2 weeks; accounts of longer persistence are rare (*25*; Technical Appendix reference *47*).

More severe clinical sequelae have increasingly been associated with Zika virus. During the ongoing outbreak in Brazil, reports of infants born with microcephaly have markedly increased (>3,800 cases; 20 cases/10,000 live births vs. 0.5/10,000 live births in previous years) (Technical Appendix reference *48*). However, concern exists that these findings may in part be artifactual, resulting from previous underreporting of cases and confounding by other risk factors for microcephaly (Technical Appendix reference *49*). Because systematic surveillance for microcephaly was not previously undertaken, the baseline rate of microcephaly in Brazil is unknown, and subsequent reports suggest that a substantial proportion of infants that reportedly have microcephaly do not actually have the condition (Technical Appendix reference *50*).

Health officials in French Polynesia have reported an apparent increase in congenital central nervous system (CNS) malformations, coinciding with the outbreak occurring during 2013–2014 (Technical Appendix reference *51*). However, this finding should be cautiously interpreted; reports included only 17 cases, and none were laboratory-confirmed Zika virus cases. In addition, the true baseline rate of such malformations before the outbreak is unknown (Technical Appendix reference *51*).

A plausible neuropathologic link between Zika virus and CNS anomalies is supported by research showing viral neurotropism in intraperitoneally infected mice (Technical Appendix reference *52*) and progression of disease in directly infected mouse brains (Technical Appendix reference *53*). One hypothesis for Zika virus’s role in CNS malformations pertains to the virus’s hijacking of autophagy during viral replication (Technical Appendix reference *54*). Some cellular proteins have a dual role in autophagy and centrosome stability; a normal number of centrosomes is important for brain development (Technical Appendix reference *54*). An increase in centrosomes in mice has been shown to result in microcephaly (Technical Appendix reference *54*). Therefore, Zika virus’s interference in autophagy has been hypothesized to lead to an increase in centrosome number and microcephaly; this potential role in malformations merits further investigation.

Severe neurologic sequelae have also been described in adults, including meningitis, meningoencephalitis, and Guillain-Barre syndrome (Technical Appendix reference *55*). A surge in Guillain-Barre syndrome cases has been observed in Brazil, Colombia, El Salvador, Suriname, Venezuela, and French Polynesia during outbreaks; however, Zika virus has been laboratory confirmed in only some of these cases (Technical Appendix reference *55*).

Nonneurologic sequelae include transient hearing loss, hypotension, and genitourinary symptoms (*11**,**36*; Technical Appendix references *56,57*). Hematospermia was reported in 2 cases (*11**,**36*). A 44-year-old man in Tahiti in whom hematospermia developed 2 weeks after symptoms of Zika virus infection was found to have replicative cultured Zika virus particles in his semen (*36*). In addition, a 36-year-old man from the United States contracted Zika virus infection while in Senegal, and subsequently, his wife was infected in the United States; this case supports sexual transmission (*11*). A second sexually acquired case was reported in Texas (Technical Appendix reference *58*).

Rare deaths have been described in patients infected with Zika virus (Technical Appendix reference *44*). Besides 1 infant death, 3 other fatalities were reported (2 from Brazil and 1 from Colombia): 1 man with lupus erythematosus, chronic corticosteroid use, rheumatoid arthritis, and alcoholism; and 2 girls 16 years of age, 1 with sickle cell disease (Technical Appendix reference *59*). (Medical history was not reported for the other girl [Technical Appendix reference *44*].)

### General Laboratory Findings

Information on laboratory findings for Zika virus infection is limited. Complete blood count is often normal; even if blood count is abnormal, changes may be nonspecific (e.g., mild lymphopenia, mild neutropenia, mild-to-moderate thrombocytopenia) (*8*; Technical Appendix references *46,60–62*). Mild elevations in inflammatory markers (C-reactive protein, fibrinogen, and ferritin), serum lactate dehydrogenase, or liver enzymes have been described (*8**,**25*; Technical Appendix reference *57*). These findings are observed in many other viral infections, including the co-circulating viruses DENV and CHIKV, so none of these laboratory alterations reliably distinguish among these infections.

### Diagnosis

Clinical evaluation alone is unreliable for a diagnosis of Zika virus infection. Because of clinical overlap with other arboviruses, diagnosis relies on laboratory testing. Evaluation for Zika virus, CHIKV, and DENV should be undertaken concurrently for all patients who have acute fever, rash, myalgia, or arthralgia after recent (previous 2 weeks) travel to an area of ongoing Zika virus transmission (Technical Appendix reference *63*). Commercial assays have been developed, including a PCR-based assay that has been approved by the Communauté Européenne (RealStar Zika Virus RT-PCR Kit 1.0, altona Diagnostics, Hamburg, Germany) and a serologic assay that has been approved by the US Food and Drug Administration for restricted use in emergency situations (Technical Appendix reference *64*). Testing has typically been performed by large reference laboratories (e.g., US CDC and US state laboratories) and universities. CDC’s typical turnaround time is 4–14 days. Appropriate tests are selected by the laboratory on the basis of clinical information provided by the requesting healthcare provider (Technical Appendix reference *65*). To coordinate sample collection, providers should contact local public health agencies before testing.

Molecular amplification (e.g., RT-PCR) on serum samples remains the most specific diagnostic approach and is the preferred testing method for Zika virus during the acute phase of illness (<7 days from symptom onset) (Technical Appendix reference *63*). In contrast, serologic testing is not recommended during the acute phase, when Zika virus IgM may be undetectable (*22*). However, molecular testing must be performed during the viremic period (*15*). Several case reports of negative RT-PCR results but positive IgM results for patients whose samples were tested at >5 days after symptom onset indicate a possible viremic period as brief as 5 days (*25**,**36*; Technical Appendix reference *61*). Consequently, testing algorithms are based on sampling relative to symptom onset, and serologic testing should be considered if samples are negative for Zika virus by RT-PCR (Technical Appendix reference *63*).

Serologic testing has limitations. Zika virus IgM and IgG are notoriously cross-reactive with those against other flaviviruses (particularly DENV), limiting specificity (*5**,**15*; Technical Appendix reference *46*). Therefore, positive serologic test results should be confirmed with testing that uses an alternative platform such as a seroneutralization assay (e.g., plaque-reduction neutralization test) (*22*). However, flaviviral cross-reactivity can also pose problems in confirmatory assays, especially for patients immunized (e.g., against YFV or Japanese encephalitis virus) or infected with another flavivirus (e.g., WNV or St. Louis encephalitis virus); presence of antibodies confounds diagnosis (Technical Appendix reference *63*).

The type of sample can also affect the probability of detection. Although diagnostic testing is performed primarily on serum or cerebrospinal fluid, the diagnostic utility of other specimen types (e.g., urine, saliva, amniotic fluid, and tissue) is being evaluated (Technical Appendix reference *63*). Urine and saliva may offer alternatives, particularly when blood collection is difficult (e.g., in children or remote locations). Viruria may persist longer than viremia. One study reported that Zika virus RNA was detected in urine up to 20 days after viremia had become undetectable (Technical Appendix reference *62*); therefore, RT-PCR testing of urine should be considered when Zika virus is clinically suspected, despite negative serum testing (*22**,**33**,**35**,**36*; Technical Appendix reference *62*). Similarly, RT-PCR conducted with saliva has been shown to increase the detection rate during the acute phase of infection but does not extend the window of detection of Zika virus RNA; consequently, blood remains the preferred sample (Technical Appendix reference *66*).

### Management and Prevention

No specific treatment or vaccine is available for Zika virus infection. Management is supportive and includes rest, fluids, antipyretics, and analgesics. Aspirin and other nonsteroidal antiinflammatory drugs should be avoided until dengue is excluded because of the risk for hemorrhage among dengue patients (Technical Appendix reference *67*).

Other general measures focus on prevention of mosquito bites, including individual protection (e.g., long pants, light-colored clothing, insect repellants, bed nets), particularly during known *Ae. aegypti* peak biting times (early morning and late afternoon) (Technical Appendix reference *68*). Community-level strategies target mosquito breeding through elimination of potential egg-laying sites (e.g., potted plant saucers, water storage units, used tires) by drying wet environments or using insecticide treatment (Technical Appendix reference *68*). Pregnant women residing in countries that are not Zika virus–endemic are advised against travel to affected countries (Technical Appendix reference *69*). Testing should be offered to all pregnant women who have traveled to areas with ongoing Zika virus transmission (Technical Appendix reference *70*). Serial fetal ultrasounds should be considered to monitor fetal anatomy and growth every 3–4 weeks in pregnant women with positive or inconclusive Zika virus test results, and the infant should be tested at birth (Technical Appendix reference *70*). Men who reside in or have traveled to an area of active Zika virus transmission and who have a pregnant partner should abstain from sexual activity or use condoms during sex; similar guidelines apply for men with a nonpregnant female sex partner who is concerned about sexual transmission of Zika virus (Technical Appendix reference *58*).

## Discussion

Zika virus has been declared a public health emergency. As many as 1.3 million persons have been affected in Brazil alone (Technical Appendix Table 2), and 20 countries or territories have reported local transmission of the virus during 2016 (Figures 2,3). Because of the ease of air travel and international trade, further spread into regions where the virus is not endemic is likely, and transmission is probable in locations with competent mosquito vectors. A robust, multifaceted response is underway that involves public health authorities, government agencies, the biomedical industry, medical practitioners, and researchers. However, uncertainty remains regarding aspects of the virus’s vectors, epidemiology, and pathogenesis. As the epidemic unfolds, evaluating incoming data critically will be necessary to separate fact from speculation.

Foremost, diagnosis remains suboptimal. Diagnostic guidelines are contingent on laboratory testing that is not widely available. Although commercial tests for Zika virus are limited in number and availability, more are in development, including prototype multiplex molecular assays that concurrently test for Zika virus, CHIKV, and DENV (M.P. Busch, pers. comm.). However, although not unique to Zika virus, laboratory infrastructure and testing capability is lacking in resource-constrained settings where Zika virus is most prevalent.

Prevention measures (specifically, vector control) are a current priority, pending advances in diagnostics; the World Health Organization and the Pan American Health Organization have issued recommendations (Technical Appendix reference *44*). In the United States, multiple factors guard against the explosive epidemic occurring throughout Central and South America. Specifically, lower rates of human crowding in urban areas, wider access to air conditioning and mosquito repellants, and waste management limit mosquitoborne transmission, which has been the case for DENV (Technical Appendix reference *71*). Nonetheless, further entomologic research is needed to define the range of Zika virus vectors and identify new areas where autochthonous transmission could take place to enable early intervention. Investment is also needed in durable control measures such as adaptable vaccine platforms for arboviruses; currently, no Zika virus vaccines are in advanced development (*9*).

Aspects of Zika virus pathogenesis remain unclear. Zika virus’s association with neurologic sequelae, including potential neuropathophysiologic mechanisms, is being actively investigated. Continued epidemiologic study, combined with research involving animal models, will offer increased insight, which could spur novel prevention strategies (*9*). If confirmed, insights into the timing of infection relative to gestational outcomes will guide policy. In the interim, new cases of Zika virus infection should be monitored for complications, particularly in babies born to mothers residing in Zika virus–affected areas. The effects of Zika virus in other vulnerable clinical subsets (e.g., those who have concurrent conditions or are immunocompromised) also merit further investigation, as does co-infection or sequential infection by co-circulating viruses.

Given reports of possible transfusion-transmitted Zika virus, the pandemic also has implications for the blood supply within Zika virus–endemic and nonendemic regions. The US Food and Drug Administration recommends 28-day deferral for blood donors with confirmed or suspected Zika virus infection (*38*). Donor screening by nucleic acid testing is being considered but will be challenging to implement because of high costs and regulatory considerations. Pathogen-reduction technology has shown efficacy for treatment of plasma (Technical Appendix reference *72*); however, absence of a licensed pathogen reduction technology for use in red cells, high incremental cost, and technical barriers render such technology an unlikely short-term solution.

Zika virus has the propensity to infect large numbers of persons with severe consequences in some cases. The epidemic has serious medical, ethical, and economic ramifications, particularly in countries where the resources for early diagnosis are lacking and potential intervention measures (e.g., contraception or termination of pregnancy) are discouraged or illegal (Technical Appendix reference *73*). Although autochthonous transmission in the United States is unlikely to match the scale of the epidemic in Central and South America, much about Zika virus and the way the pandemic will evolve are unknown. Continued vigilance is warranted, along with a concerted effort toward improving our understanding, management, and prevention of this emerging pathogen.

**Technical Appendix.** Additional references and tables summarizing findings of literature search regarding current knowledge of Zika virus.
