# Supplementary material for: A Literature Review of Zika Virus
Source: Emerg Infect Dis. 2016 Jul;22(7):1185–92. doi: 10.3201/eid2207.151990 (PMC4918175; doi:10.3201/eid2207.151990)
Supplement: Supplementary file 1 — Technical Appendix. Additional references and tables summarizing findings of literature search regarding current knowledge of Zika virus. [file 15-1990-Techapp-s1.pdf]

Article DOI: http://dx.doi.org/10.3201/eid2207.151990

# A Literature Review of Zika Virus

## Technical Appendix

**Technical Appendix Table 1.** Seroprevalence surveys of Zika virus infection by location, January 1, 1952–February 10, 2016*

| Continent/country | % Seropositivity (details) | Years survey performed | Laboratory assessment method | Ref |
|---|---|---|---|---|
| Africa | | | | |
| Kenya | 17.6 (476/2,698 serum samples) | 1966–1968 | HI only | (*74*) |
| Nigeria† | a) 31.2 (59/189 by HI; 40.3 (121/300 by neutralization) b) 56.2 (150/267) c) 11.5 (15/131 by HI, 1965 and 1970); 12.2 (18/147 by HI and neutralization, 1970–1971) | a) 1971–1975 (b) 1980 c) 1965, 1970–1971 | a) HI and/or neutralization b) HA and HI only c) HI or HI followed by neutralization | a) (*75*) b) (*76*) c) (*77*) |
| Côte d'Ivoire | 47.6 (20/42) | 1999 | IgG by ELISA only | (*78*) |
| Senegal | 6.7 (57/852) | 1988, 1990 | IgM by ELISA only | (*79*) |
| The Gambia | 86.0 (37/43 by HI); 23.8 (10/28 by CF) | 1979 | HI or CF | (*80*) |
| Uganda† | a) 12 (261 patients tested)‡ b) 6.1 (8/132) c) 6.1 (6/99) | (a) 1945–1948 (b) 1984 (c) Not specified | (a) Viral neutralization (b) HI only (c) Not specified§ | a) (*81*) b) (*82*) c) (*52*) |
| Tanzania | 17 (36 patients tested)‡ | 1945–1948 | Viral neutralization | (*81*) |
| Zambia | 6.0 (217/3,625) | Unspecified | Unspecified¶ | (*83*) |
| Asia | | | | |
| Borneo | 44.1 (49/111) | 1996–1997 | "Plasma samples were tested for neutralizing antibody…sera were screened at a 1:10 dilution. Endpoint titrations were conducted for samples that tested positive (i.e., titer >10)." | (*84*) |
| Pakistan | 2.3 (1/43) | 1979, 1980 | Initial screen for antibodies (not further specified), followed by CF | (*85*) |
| Oceania | | | | |
| Yap Island, Micronesia | 74.3 (414/557) | 2007 | IgM by ELISA only | (*5*) |

*Summary data of all seroprevalence surveys of Zika virus antibodies in humans, reported in peer-reviewed literature, 1952–2016. Publications for which the abstract only was accessible or that did not provide complete data were excluded. CF, complement fixation; HA, hemagglutination assay; HI, hemagglutination-inhibition; Ref, Technical Appendix reference number.
† Multiple studies were found for Nigeria and Uganda; these studies are indicated separately with a, b, c.
‡ Study for Uganda and United Republic of Tanzania reported only numbers of patients tested and whole percentages of seropositive patients (*81*).
§Methodology information provided in primary source: "Human sera tested for presence of Zika virus antibody...Only those sera which were found to neutralize 100 or more LD50 have been considered as positive" (*52*).
¶Methodology information provided in primary source: "Serum was assessed for IgG and IgM antibodies" (*83*).

**Technical Appendix Table 2.** Outbreaks and case reports of laboratory-confirmed Zika virus infection by location, January 1, 1952–February 10, 2016*

| Continent/country | No. cases | Years | Ref | Comments |
|---|---|---|---|---|
| Africa | | | | |
| Cape Verde | 17 | 2015 | (*86*) | 15 IgM positive only; 2 positive by reverse transcription PCR |
| Gabon | 5 | 2007–2010 | (*23*) | |
| Nigeria | 5 | 1952, 1971–1975 | (*2,75*) | |
| Uganda | 1 | Not specified | (*40*) | |
| Asia | | | | |
| Cambodia | 1 | 2010 | (*56*) | |
| Indonesia | 7 | 1977–1978 | (*24*) | |
| Japan | 3 | 2013–2014 | (*87,88*) | Imported cases (ex-Thailand, ex-French Polynesia) |
| Thailand | 7 | 2012–2014 | (*89*) | |
| Philippines | 1 | 2012 | (*67*) | |
| Caribbean | | | | |
| Dominican Republic | 10 | 2016 | (*90*) | 8 local cases; 2 imported cases (ex-El Salvador) |
| Guadeloupe | 10 | 2016 | (*91*) | |
| Jamaica | 1 | 2016 | (*92*) | |
| St. Croix, US Virgin Islands | 1 | 2016 | (*93*) | |
| Central America | | | | |
| Costa Rica | 2 | 2016 | (*94*) | 1 case imported (ex-Colombia); other case not specified |
| Mexico | 3 | 2015 | (*95*) | 2 local cases; 1 imported case (ex-Colombia) |
| Nicaragua | 2 | 2016 | (*91,96*) | |
| Europe | | | | |
| Denmark | 1 | 2016 | (*91,97*) | Imported case (ex-Mexico and Brazil) |
| Finland | 1 | 2015 | (*3*) | Imported case (ex-Maldives) |
| France | 1 | 2013 | (*98*) | Imported case (ex-French Polynesia) |
| Germany | 4 | 2013, 2014, 2016 | (*57,99,100*) | Imported cases (ex-Haiti, ex-Malaysian Borneo, ex-Thailand) |
| Italy | 3 | 2013–2014, 2015 | (*60,101*) | |
| Netherlands | 13 | 2015 | (*102,103*) | Imported cases (ex-Suriname [1]; country of import unspecified [12]) |
| Norway | 1 | 2013 | (61) | Imported case (ex-Tahiti) |
| Portugal | 5 | 2016 | (*91,104*) | Imported cases (ex-Brazil) |
| Spain | 7 | 2016 | (*105*) | Imported cases (country of import unspecified) |
| Sweden | 1 | 2015 | (*91,106*) | Imported case (country of import unspecified) |
| United Kingdom | 6 | 2014–2016 | (*107*) | Imported cases (ex-Colombia [3], ex-Cook-Islands [1], ex-Guyana/Suriname [1], and ex-Mexico/Venezuela [1]) |
| North America | | | | |
| Canada | 2 | 2013, 2016 | (*14,46*) | Imported cases (ex-Thailand, ex-El Salvador) |
| United States | 56 | 2008, 2013, 2016 | (*10,12,36,68*) | Cases reported in Arkansas (1), California (2), Colorado (3), Delaware (1), District of Columbia (3), Florida (16), Georgia (1), Hawaii (4), Illinois (3), Massachusetts (2), Minnesota (1), Nebraska (2), New Jersey (1), New York (1), Ohio (1), Oregon (1), Pennsylvania (2), Virginia (1), Texas (10). All were imported cases, except for 2 autochthonous sexually-acquired cases, including 1 in Colorado and 1 in Texas. Reported locations of import include Senegal, French Polynesia, South America, Easter Island, and Hawaii. |
| Oceania | | | | |
| Australia | 3 | 2014 (1 case); year unspecified (2 cases) | (*39,108,109*) | Imported cases (ex-Indonesia [2], ex-Cook Islands [1]) |
| Cook Islands | 50 | 2014 | (*6*) | 932 additional suspected cases |
| Easter Island | 53 | 2014 | (*7,110,111*) | One imported case (ex-Tahiti); 40 additional suspected cases |

| Continent/country | No. cases | Years | Ref | Comments |
|---|---|---|---|---|
| Fiji | 1 | 2015 | (*112*) | |
| French Polynesia | 383 | 2013–2014 | (*14*) | 32,000 additional suspected cases |
| New Caledonia | 1,385 | 2014 | (*8*) | |
| New Zealand | 67 | 2002–2015, 2016 | (*113*) | Imported cases (ex-Cook Islands [27],ex-Vanuatu [1], ex-Tonga [4], ex-Samoa [4], unspecified country of import [1]) |
| Samoa | 1 (at least) | 2016 | (*91,114*) | |
| Solomon Islands | 4 | 2015 | (*115*) | |
| Tonga | 1 | 2016 | (*113*) | |
| Vanuatu | 2 | 2015 | (*115*) | |
| Yap, Federated States of Micronesia | 108 | 2007 | (*5*) | 919 additional suspected cases |
| South America | | | | |
| Barbados | 3 | 2016 | (*116*) | |
| Bolivia | 1 | 2016 | (*117*) | |
| Brazil | 69 | 2015–2016 | (*14,54,118–121*) | 440,000–1,300,000 estimated total cases |
| Chile (Mainland) | 3 | 2016 | (*122*) | Imported cases (ex-Venezuela [1], ex-Colombia [1], ex-Brazil [1]) |
| Colombia | 1,050 | 2015 | (*123*) | |
| Curacao | 1 | 2016 | (*91*) | |
| Ecuador | 6 | 2016 | (*116*) | 2 local cases; 4 imported cases (ex-Colombia [3], ex-Venezuela [1]). |
| El Salvador | 3 | 2015–2016 | (*124,125*) | 3,836 additional suspected cases |
| French Guiana | 59 | 2016 | (*91*) | |
| Guatemala | 1 | 2015 | (*124*) | |
| Guyana | 164 | 2016 | (*91*) | |
| Haiti | 5 | 2016 | (*100*) | |
| Honduras | 2 | 2015 | (*126*) | |
| Martinique | 2 | 2016 | (*91*) | |
| Panama | 4 | 2015 | (*127*) | |
| Paraguay | 6 | 2015 | (*128*) | |
| Puerto Rico | 9 | 2016 | (*10*) | 1 imported case; 8 local cases |
| Saint Martin | 1 | 2016 | (*129*) | |
| Suriname | 6 | 2015 | (*130*) | |
| Venezuela | 4 | 2015 | (*131*) | |

*Data represent outbreaks and case reports for all reported laboratory-confirmed cases of Zika virus infection (confirmed by serology and/or RT-PCR, or mode of laboratory testing not specified), including those reported in peer-reviewed literature; public health agency Web sites, bulletins, and broadcasts; and media reports, January 1, 1952–February 10, 2016. Ref, Technical Appendix reference number; ex-, exported from.

**Technical Appendix Table 3.** Symptoms and sequelae of 195 symptomatic patients with confirmed Zika virus infection reported in Peer-reviewed literature for 1964–2016*

| Symptom | No. of patients | % Total patients |
|---|---|---|
| Rash | 131 | 67.2 |
| Fever | 124 | 63.6 |
| Arthralgia | 56 | 28.7 |
| Myalgia | 46 | 23.6 |
| Headache | 42 | 21.5 |
| Conjunctivitis | 40 | 20.5 |
| Retroorbital pain | 22 | 11.3 |
| Edema | 19 | 9.7 |
| Pruritus | 15 | 7.7 |
| Fatigue/asthenia | 14 | 7.2 |

*Data represent reports that included clinical symptoms and sequelae for all symptomatic patients with laboratory-confirmed Zika virus infection (confirmed by serologic testing or RT-PCR, or mode of laboratory testing not specified), with a complete and detailed account of symptoms reported in peer-reviewed literature for January 1, 1964–February 3, 2016 (age range of patients was 4 days–76 years). Reports only for this period included symptoms and therefore met criteria for inclusion in the table. Patients may have experienced ≥1 symptom. Less prevalent reported symptoms and sequelae (<5% of patients with reported symptoms) include microcephaly, cerebral calcifications, Guillain-Barré syndrome, prostatitis, hematospermia, hematuria, hypotension, gingival bleeding, hearing loss, pruritus, malaise, diarrhea, nausea/vomiting, constipation, edema, sore throat, cough, stomach pain, anorexia, lightheadedness, dizziness, chills, oral aphthous ulcers, photophobia, lymphadenopathy, rhinorrhea, and burning sensation of palms/soles.
References: (*3,7,8,11,23–25,34,36,37,39–41,46,47,56,57,60–62,68,75,87,88,99,101,108,109*).

## References

1. Dick GW, Kitchen SF, Haddow AJ. Zika virus. I. Isolations and serological specificity. Trans R Soc Trop Med Hyg. 1952;46:509–20. http://dx.doi.org/10.1016/0035-9203(52)90042-4

2. MacNamara FN. Zika virus: a report on three cases of human infection during an epidemic of jaundice in Nigeria. Trans R Soc Trop Med Hyg. 1954;48:139–45. http://dx.doi.org/10.1016/0035-9203(54)90006-1

3. Korhonen EM, Huhtamo E, Smura T, Kallio-Kokko H, Raassina M, Vapalahti O. Zika virus infection in a traveller returning from the Maldives, June 2015. Euro Surveill. 2016;21:30107. http://dx.doi.org/10.2807/1560-7917.ES.2016.21.2.30107

4. Baba SS, Fagbami AH, Ojeh CK. Preliminary studies on the use of solid-phase immunosorbent techniques for the rapid detection of Wesselsbron virus (WSLV) IgM by haemagglutination-inhibition. Comp Immunol Microbiol Infect Dis. 1999;22:71–9. http://dx.doi.org/10.1016/S0147-9571(98)00003-4

5. Duffy MR, Chen TH, Hancock WT, Powers AM, Kool JL, Lanciotti RS, et al. Zika virus outbreak on Yap Island, Federated States of Micronesia. N Engl J Med. 2009;360:2536–43. http://dx.doi.org/10.1056/NEJMoa0805715

6. Roth A, Mercier A, Lepers C, Hoy D, Duituturaga S, Benyon E, et al. Concurrent outbreaks of dengue, chikungunya and Zika virus infections - an unprecedented epidemic wave of mosquito-borne viruses in the Pacific 2012-2014. Euro Surveill. 2014;19:20929. http://dx.doi.org/10.2807/1560-7917.ES2014.19.41.20929

7. Tognarelli J, Ulloa S, Villagra E, Lagos J, Aguayo C, Fasce R, et al. A report on the outbreak of Zika virus on Easter Island, South Pacific, 2014. Arch Virol. 2016;161:665–8. http://dx.doi.org/10.1007/s00705-015-2695-5

8. Dupont-Rouzeyrol M, O'Connor O, Calvez E, Daurès M, John M, Grangeon JP, et al. Co-infection with Zika and dengue viruses in 2 patients, New Caledonia, 2014. Emerg Infect Dis. 2015;21:381–2. http://dx.doi.org/10.3201/eid2102.141553

9. Fauci AS, Morens DM. Zika virus in the Americas—yet another arbovirus threat. N Engl J Med. 2016;374:601–4. http://dx.doi.org/10.1056/NEJMp1600297

10. Centers for Disease Control and Prevention. Zika virus disease in the United States, 2015–2016. 2016 Feb 10 [cited 2016 Feb 17]. http://www.cdc.gov/zika/geo/united-states.html

11. Foy BD, Kobylinski KC, Chilson Foy JL, Blitvich BJ, Travassos da Rosa A, Haddow AD, et al. Probable non-vector-borne transmission of Zika virus, Colorado, USA. Emerg Infect Dis. 2011;17:880–2. http://dx.doi.org/10.3201/eid1705.101939

12. Dallas County Health and Human Services. DCHHS reports first Zika virus case in Dallas County acquired through sexual transmission. 2016 Feb 2 [cited 2016 Feb 8]. http://www.dallascounty.org/department/hhs/press/documents/PR2-2-16DCHHSReportsFirstCaseofZikaVirusThroughSexualTransmission.pdf

13. Hennessey M, Fischer M, Staples JE. Zika virus spreads to new areas—region of the Americas, May 2015–January 2016. MMWR Morb Mortal Wkly Rep. 2016;65:55–8. http://dx.doi.org/10.15585/mmwr.mm6503e1

14. European Centre for Disease Prevention and Control. Zika virus disease epidemic: potential association with microcephaly and Guillain-Barré syndrome (first update). 2016 Jan 21 [cited 2016 Feb 3]. http://ecdc.europa.eu/en/publications/Publications/rapid-risk-assessment-zika-virus-first-update-jan-2016.pdf

15. Lanciotti RS, Kosoy OL, Laven JJ, Velez JO, Lambert AJ, Johnson AJ, et al. Genetic and serologic properties of Zika virus associated with an epidemic, Yap State, Micronesia, 2007. Emerg Infect Dis. 2008;14:1232–9. http://dx.doi.org/10.3201/eid1408.080287

16. Kuno G, Chang GJ. Full-length sequencing and genomic characterization of Bagaza, Kedougou, and Zika viruses. Arch Virol. 2007;152:687–96. http://dx.doi.org/10.1007/s00705-006-0903-z

17. Gatherer D, Kohl A. Zika virus: a previously slow pandemic spreads rapidly through the Americas. J Gen Virol. 2016;97:269–73. http://dx.doi.org/10.1099/jgv.0.000381

18. Faye O, Freire CC, Iamarino A, Faye O, de Oliveira JV, Diallo M, et al. Molecular evolution of Zika virus during its emergence in the 20(th) century. PLoS Negl Trop Dis. 2014;8:e2636. http://dx.doi.org/10.1371/journal.pntd.0002636

19. Hamel R, Dejarnac O, Wichit S, Ekchariyawat P, Neyret A, Luplertlop N, et al. Biology of Zika virus infection in human skin cells. J Virol. 2015;89:8880–96. http://dx.doi.org/10.1128/JVI.00354-15

20. Buckley A, Gould EA. Detection of virus-specific antigen in the nuclei or nucleoli of cells infected with Zika or Langat virus. J Gen Virol. 1988;69:1913–20. http://dx.doi.org/10.1099/0022-1317-69-8-1913

21. Marchette NJ, Garcia R, Rudnick A. Isolation of Zika virus from *Aedes aegypti* mosquitoes in Malaysia. Am J Trop Med Hyg. 1969;18:411–5.

22. Hayes EB. Zika virus outside Africa. Emerg Infect Dis. 2009;15:1347–50. http://dx.doi.org/10.3201/eid1509.090442

23. Grard G, Caron M, Mombo IM, Nkoghe D, Mboui Ondo S, Jiolle D, et al. Zika virus in Gabon (Central Africa)—2007: a new threat from *Aedes albopictus?* PLoS Negl Trop Dis. 2014;8:e2681. http://dx.doi.org/10.1371/journal.pntd.0002681

24. Olson JG, Ksiazek TG, Suhandiman, Triwibowo. Zika virus, a cause of fever in Central Java, Indonesia. Trans R Soc Trop Med Hyg. 1981;75:389–93. http://dx.doi.org/10.1016/0035-9203(81)90100-0

25. Oehler E, Watrin L, Larre P, Leparc-Goffart I, Lastere S, Valour F, et al. Zika virus infection complicated by Guillain-Barre syndrome—case report, French Polynesia, December 2013. Euro Surveill. 2014;19:20720. http://dx.doi.org/10.2807/1560-7917.ES2014.19.9.20720

26. Diallo D, Sall AA, Diagne CT, Faye O, Faye O, Ba Y, et al. Zika virus emergence in mosquitoes in southeastern Senegal, 2011. PLoS One. 2014;9:e109442. http://dx.doi.org/10.1371/journal.pone.0109442

27. Boorman JP, Porterfield JS. A simple technique for infection of mosquitoes with viruses; transmission of Zika virus. Trans R Soc Trop Med Hyg. 1956;50:238–42. http://dx.doi.org/10.1016/0035-9203(56)90029-3

28. Musso D, Nilles EJ, Cao-Lormeau VM. Rapid spread of emerging Zika virus in the Pacific area. Clin Microbiol Infect. 2014;20:O595–6. http://dx.doi.org/10.1111/1469-0691.12707

29. Ledermann JP, Guillaumot L, Yug L, Saweyog SC, Tided M, Machieng P, et al. *Aedes hensilli* as a potential vector of Chikungunya and Zika viruses. PLoS Negl Trop Dis. 2014;8:e3188. http://dx.doi.org/10.1371/journal.pntd.0003188

30. Kraemer MU, Sinka ME, Duda KA, Mylne A, Shearer FM, Brady OJ, et al. The global compendium of *Aedes aegypti* and *Ae. albopictus* occurrence. Sci Data. 2015;2:150035. http://dx.doi.org/10.1038/sdata.2015.35

31. Gaffigan TVWR, Pecor JE, Stoffer JA, Anderson T. Systematic catalog of culicidae. Suitland (MD): Smithsonian Institution; Silver Spring (MD): Walter Reed Army Institute of Research; 2016 [cited 2016 Feb 10]. http://mosquitocatalog.org/

32. Ioos S, Mallet HP, Leparc Goffart I, Gauthier V, Cardoso T, Herida M. Current Zika virus epidemiology and recent epidemics. Med Mal Infect. 2014;44:302–7. http://dx.doi.org/10.1016/j.medmal.2014.04.008

33. Haddow AD, Schuh AJ, Yasuda CY, Kasper MR, Heang V, Huy R, et al. Genetic characterization of Zika virus strains: geographic expansion of the Asian lineage. PLoS Negl Trop Dis. 2012;6:e1477. http://dx.doi.org/10.1371/journal.pntd.0001477

34. Oliveira Melo AS, Malinger G, Ximenes R, Szejnfeld PO, Alves Sampaio S, Bispo de Filippis AM. Zika virus intrauterine infection causes fetal brain abnormality and microcephaly: tip of the iceberg? Ultrasound Obstet Gynecol. 2016;47:6–7. http://dx.doi.org/10.1002/uog.15831

35. Besnard M, Lastere S, Teissier A, Cao-Lormeau V, Musso D. Evidence of perinatal transmission of Zika virus, French Polynesia, December 2013 and February 2014. Euro Surveill. 2014;19:20751. http://dx.doi.org/10.2807/1560-7917.ES2014.19.13.20751

36. Musso D, Roche C, Robin E, Nhan T, Teissier A, Cao-Lormeau VM. Potential sexual transmission of Zika virus. Emerg Infect Dis. 2015;21:359–61. http://dx.doi.org/10.3201/eid2102.141363

37. Musso D, Nhan T, Robin E, Roche C, Bierlaire D, Zisou K, et al. Potential for Zika virus transmission through blood transfusion demonstrated during an outbreak in French Polynesia, November 2013 to February 2014. Euro Surveill. 2014;19:20761. http://dx.doi.org/10.2807/1560-7917.ES2014.19.14.20761

38. US Food and Drug Administration. Recommendations for donor screening, deferral, and product management to reduce the risk of transfusion transmission of Zika virus, recommendations for industry. 2016 Feb 16 [cited 2016 Feb 16]. http://www.fda.gov/downloads/BiologicsBloodVaccines/GuidanceComplianceRegulatoryInformation/Guidances/Blood/UCM486360.pdf

39. Leung GH, Baird RW, Druce J, Anstey NM. Zika virus infection in Australia following a monkey bite in Indonesia. Southeast Asian J Trop Med Public Health. 2015;46:460–4.

40. Simpson DI. Zika virus infection in man. Trans R Soc Trop Med Hyg. 1964;58:335–8. http://dx.doi.org/10.1016/0035-9203(64)90201-9

41. Filipe AR, Martins CM, Rocha H. Laboratory infection with Zika virus after vaccination against yellow fever. Arch Gesamte Virusforsch. 1973;43:315–9. http://dx.doi.org/10.1007/BF01556147

42. Martines RB, Bhatnagar J, Keating MK, Silva-Flannery L, Muehlenbachs A, Gary J, et al. Notes from the field: evidence of Zika virus infection in brain and placental tissues from two congenitally infected newborns and two fetal losses—Brazil, 2015. MMWR Morb Mortal Wkly Rep. 2016;65:159–60. http://dx.doi.org/10.15585/mmwr.mm6506e1

43. European Centre for Disease Prevention and Control. Rapid risk assessment. Zika virus epidemic in the Americas: potential association with microcephaly and Guillain-Barré syndrome. 2015 Dec 10. [cited 2016 Feb 3]. http://ecdc.europa.eu/en/publications/Publications/zika-virus-americas-association-with-microcephaly-rapid-risk-assessment.pdf

44. Pan American Health Organization; World Health Organization. Epidemiological alert. Neurological syndrome, congenital malformations, and Zika virus infection. Implications for public health in the Americas. 2015 Dec 1 [cited 2016 Feb 10]. 2015-dec-1-cha-epi-alert-zika-neuro-syndrome.pdf

45. Mlakar J, Korva M, Tul N, Popović M, Poljšak-Prijatelj M, Mraz J, et al. Zika virus associated with microcephaly. N Engl J Med. 2016;374:951–8. http://dx.doi.org/10.1056/NEJMoa1600651

46. Fonseca K, Meatherall B, Zarra D, Drebot M, MacDonald J, Pabbaraju K, et al. First case of Zika virus infection in a returning Canadian traveler. Am J Trop Med Hyg. 2014;91:1035–8. http://dx.doi.org/10.4269/ajtmh.14-0151

47. Zanluca C, Melo VC, Mosimann AL, Santos GI, Santos CN, Luz K. First report of autochthonous transmission of Zika virus in Brazil. Mem Inst Oswaldo Cruz. 2015;110:569–72. http://dx.doi.org/10.1590/0074-02760150192

48. Schuler-Faccini L, Ribeiro EM, Feitosa IM, Horovitz DD, Cavalcanti DP, Pessoa A, et al.; Brazilian Medical Genetics Society–Zika Embryopathy Task Force. Possible association between Zika virus infection and microcephaly—Brazil, 2015. MMWR Morb Mortal Wkly Rep. 2016;65:59–62. http://dx.doi.org/10.15585/mmwr.mm6503e2

49. Simmins CH Jr. Establishing base levels of microcephaly in Brazil prior to the arrival of Zika viral illnesses. Bull World Health Organ. 2016 [cited 2016 Feb 10]. Epub 2016 Feb 8.

50. World Health Organization. Zika situation report: Zika and potential complications, 12 Feburary 2016. 2016 Feb 12 [cited 2016 Feb 12]. http://www.who.int/emergencies/zika-virus/situation-report/who-zika-situation-report-12-02-2016.pdf?ua=1

51. European Centre for Disease Prevention and Control. Rapid risk assessment: microcephaly in Brazil potentially linked to the Zika virus epidemic. 2015 Nov 24 [cited 2016 Feb 3]. http://ecdc.europa.eu/en/publications/Publications/zika-microcephaly-Brazil-rapid-risk-assessment-Nov-2015.pdf

52. Dick GW. Zika virus. II. Pathogenicity and physical properties. Trans R Soc Trop Med Hyg. 1952;46:521–34. http://dx.doi.org/10.1016/0035-9203(52)90043-6

53. Bell TM, Field EJ, Narang HK. Zika virus infection of the central nervous system of mice. Arch Gesamte Virusforsch. 1971;35:183–93. http://dx.doi.org/10.1007/BF01249709

54. Tetro JA. Zika and microcephaly: causation, correlation, or coincidence? Microbes Infect. 2016;18:167–8. http://dx.doi.org/10.1016/j.micinf.2015.12.010

55. World Health Organization. Zika situation report: neurological syndrome and congenital anomalies. 2016 Feb 5 [cited 2016 Feb 8]. http://www.who.int/emergencies/zika-virus/situation-report/5-february-2016/en/

56. Heang V, Yasuda CY, Sovann L, Haddow AD, Travassos da Rosa AP, Tesh RB, et al. Zika virus infection, Cambodia, 2010. Emerg Infect Dis. 2012;18:349–51. http://dx.doi.org/10.3201/eid1802.111224

57. Tappe D, Nachtigall S, Kapaun A, Schnitzler P, Günther S, Schmidt-Chanasit J. Acute Zika virus infection after travel to Malaysian Borneo, September 2014. Emerg Infect Dis. 2015;21:911–3. http://dx.doi.org/10.3201/eid2105.141960

58. Oster AM, Brooks JT, Stryker JE, Kachur RE, Mead P, Pesik NT, et al. Interim guidelines for prevention of sexual transmission of Zika Virus—United States, 2016. MMWR Morb Mortal Wkly Rep. 2016;65:120–1. http://dx.doi.org/10.15585/mmwr.mm6505e1

59. Arzuza-Ortega LPA, Pérez-Tatis G, López-García H, Parra E, Pardo-Herrera LC, Rico-Turca AM, et al. Fatal Zika virus infection in girl with sickle cell disease, Colombia [letter]. Emerg Infect Dis. 2016 May [*date cited*]. http://dx.doi.org/10.3201/eid2205.151934

60. Zammarchi L, Stella G, Mantella A, Bartolozzi D, Tappe D, Günther S, et al. Zika virus infections imported to Italy: clinical, immunological and virological findings, and public health implications. J Clin Virol. 2015;63:32–5. http://dx.doi.org/10.1016/j.jcv.2014.12.005

61. Wæhre T, Maagard A, Tappe D, Cadar D, Schmidt-Chanasit J. Zika virus infection after travel to Tahiti, December 2013. Emerg Infect Dis. 2014;20:1412–4. http://dx.doi.org/10.3201/eid2008.140302

62. Gourinat AC, O'Connor O, Calvez E, Goarant C, Dupont-Rouzeyrol M. Detection of Zika virus in urine. Emerg Infect Dis. 2015;21:84–6. http://dx.doi.org/10.3201/eid2101.140894

63. Centers for Disease Control and Prevention. Updated diagnostic testing for Zika, chikungunya, and dengue viruses in US public health laboratories. 2016 Feb 7 [cited 2016 Feb 8]. http://www.cdc.gov/zika/pdfs/denvchikvzikv-testing-algorithm.pdf

64. Centers for Disease Control and Prevention. New CDC laboratory test for Zika virus authorized for emergency use by FDA. 2016 Feb 26 [cited 2016 Feb 26]. http://www.cdc.gov/media/releases/2016/s0226-laboratory-test-for-zika-virus.html

65. Centers for Disease Control and Prevention. Zika virus: diagnostic testing. 2016 Feb 11 [cited 2016 Feb 11]. http://www.cdc.gov/zika/hc-providers/diagnostic.html

66. Musso D, Roche C, Nhan TX, Robin E, Teissier A, Cao-Lormeau VM. Detection of Zika virus in saliva. J Clin Virol. 2015;68:53–5. http://dx.doi.org/10.1016/j.jcv.2015.04.021

67. Alera MT, Hermann L, Tac-An IA, Klungthong C, Rutvisuttinunt W, Manasatienkij W, et al. Zika virus infection, Philippines, 2012. Emerg Infect Dis. 2015;21:722–4. http://dx.doi.org/10.3201/eid2104.141707

68. Summers DJ, Acosta RW, Acosta AM. Zika virus in an American recreational traveler. J Travel Med. 2015;22:338–40. http://dx.doi.org/10.1111/jtm.12208

69. Petersen EE, Staples JE, Meaney-Delman D, Fischer M, Ellington SR, Callaghan WM, et al. Interim guidelines for pregnant women during a Zika virus outbreak—United States, 2016. MMWR Morb Mortal Wkly Rep. 2016;65:30–3. http://dx.doi.org/10.15585/mmwr.mm6502e1

70. Oduyebo T, Petersen EE, Rasmussen SA, Mead PS, Meaney-Delman D, Renquist CM, et al. Update: interim guidelines for health care providers caring for pregnant women and women of reproductive age with possible Zika virus exposure—United States, 2016. MMWR Morb Mortal Wkly Rep. 2016;65:122–7. http://dx.doi.org/10.15585/mmwr.mm6505e2

71. Reiter P, Lathrop S, Bunning M, Biggerstaff B, Singer D, Tiwari T, et al. Texas lifestyle limits transmission of dengue virus. Emerg Infect Dis. 2003;9:86–9. http://dx.doi.org/10.3201/eid0901.020220

72. Aubry M, Richard V, Green J, Broult J, Musso D. Inactivation of Zika virus in plasma with amotosalen and ultraviolet A illumination. Transfusion. 2016;56:33–40. http://dx.doi.org/10.1111/trf.13271

73. News BBC. Q&A: Abortion rules in Zika-affected countries. 2016 Jan 29 [cited 2016 Feb 10]. http://www.bbc.com/news/world-latin-america-35438404

74. Geser A, Henderson BE, Christensen S. A multipurpose serological survey in Kenya. 2. Results of arbovirus serological tests. Bull World Health Organ. 1970;43:539–52.

75. Fagbami AH. Zika virus infections in Nigeria: virological and seroepidemiological investigations in Oyo State. J Hyg (Lond). 1979;83:213–9. http://dx.doi.org/10.1017/S0022172400025997

76. Adekolu-John EO, Fagbami AH. Arthropod-borne virus antibodies in sera of residents of Kainji Lake Basin, Nigeria 1980. Trans R Soc Trop Med Hyg. 1983;77:149–51. http://dx.doi.org/10.1016/0035-9203(83)90053-6

77. Monath TP, Wilson DC, Casals J. The 1970 yellow fever epidemic in Okwoga District, Benue Plateau State, Nigeria. 3. Serological responses in persons with and without pre-existing heterologous group B immunity. Bull World Health Organ. 1973;49:235–44.

78. Akoua-Koffi C, Diarrassouba S, Bénié VB, Ngbichi JM, Bozoua T, Bosson A, et al. [Investigation surrounding a fatal case of yellow fever in Côte d'Ivoire in 1999]. Bull Soc Pathol Exot. 2001;94:227–30.

79. Monlun E, Zeller H, Le Guenno B, Traoré-Lamizana M, Hervy JP, Adam F, et al. [Surveillance of the circulation of arbovirus of medical interest in the region of eastern Senegal]. Bull Soc Pathol Exot. 1993;86:21–8.

80. Monath TP, Craven RB, Muth DJ, Trautt CJ, Calisher CH, Fitzgerald SA. Limitations of the complement-fixation test for distinguishing naturally acquired from vaccine-induced yellow fever infection in flavivirus-hyperendemic areas. Am J Trop Med Hyg. 1980;29:624–34.

81. Smithburn KC. Neutralizing antibodies against certain recently isolated viruses in the sera of human beings residing in East Africa. J Immunol. 1952;69:223–34.

82. Rodhain F, Gonzalez JP, Mercier E, Helynck B, Larouze B, Hannoun C. Arbovirus infections and viral haemorrhagic fevers in Uganda: a serological survey in Karamoja district, 1984. Trans R Soc Trop Med Hyg. 1989;83:851–4. http://dx.doi.org/10.1016/0035-9203(89)90352-0

83. Babaniyi OA, Mwaba P, Mulenga D, Monze M, Songolo P, Mazaba-Liwewe ML, et al. Risk assessment for yellow Fever in Western and north-Western provinces of Zambia. J Glob Infect Dis. 2015;7:11–7. http://dx.doi.org/10.4103/0974-777X.150884

84. Wolfe ND, Kilbourn AM, Karesh WB, Rahman HA, Bosi EJ, Cropp BC, et al. Sylvatic transmission of arboviruses among Bornean orangutans. Am J Trop Med Hyg. 2001;64:310–6.

85. Darwish MA, Hoogstraal H, Roberts TJ, Ahmed IP, Omar F. A sero-epidemiological survey for certain arboviruses (Togaviridae) in Pakistan. Trans R Soc Trop Med Hyg. 1983;77:442–5. http://dx.doi.org/10.1016/0035-9203(83)90106-2

86. World Health Organization. Zika virus infection—Cape Verde. 2015 Dec 21 [cited 2016 Feb 3]. http://www.who.int/csr/don/21-december-2015-zika-cape-verde/en/

87. Kutsuna S, Kato Y, Takasaki T, Moi M, Kotaki A, Uemura H, et al. Two cases of Zika fever imported from French Polynesia to Japan, December 2013 to January 2014 [corrected]. Euro Surveill. 2014;19:20683. http://dx.doi.org/10.2807/1560-7917.ES2014.19.4.20683

88. Shinohara K, Kutsuna S, Takasaki T, Moi ML, Ikeda M, Kotaki A, et al. Zika fever imported from Thailand to Japan, and diagnosed by PCR in the urines. J Travel Med. 2016;23:tav011. http://dx.doi.org/10.1093/jtm/tav011

89. Buathong R, Hermann L, Thaisomboonsuk B, Rutvisuttinunt W, Klungthong C, Chinnawirotpisan P, et al. Detection of Zika Virus Infection in Thailand, 2012-2014. Am J Trop Med Hyg. 2015;93:380–3. http://dx.doi.org/10.4269/ajtmh.15-0022

90. World Health Organization. Zika virus infection—Dominican Republic. 2016 Jan 27 [cited 2016 Feb 3]. http://www.who.int/csr/don/27-january-2016-zika-dominican-republic/en/

91. European Centre for Disease Prevention and Control. Epidemiological update: outbreaks of Zika virus and complications potentially linked to the Zika virus infection. 2016 Feb 5 [cited 2016 Feb 5].

92. Jamaica Ministry of Health. Jamaica confirms first case of the Zika virus. 2016 Jan 30 [cited 2016 Feb 3]. http://moh.gov.jm/jamaica-confirms-first-case-of-the-zika-virus/

93. World Health Organization. Zika virus infection—United States of America–United States Virgin Islands. 2016 Jan 29 [cited 2016 Feb 3]. http://www.who.int/csr/don/29-january-2016-zika-usa/en/

94. Dyer Z. First Zika virus case reported in Costa Rica. The Tico Times. 2016 Jan 26 [cited 2016 Feb 10]. http://www.ticotimes.net/2016/01/26/first-zika-virus-case-reported-costa-rica

95. World Health Organization. Zika virus infection—Mexico. 2015 Dec 3 [cited 2016 Feb 3]. http://www.who.int/csr/don/03-december-2015-zika-mexico/en/

96. Decavele J. Honduras, Nicaragua report first cases of Zika in pregnant women. Reuters. 2016 Feb 4 [cited 2016 Feb 8]. http://www.reuters.com/article/us-health-zika-nicaragua-idUSKCN0VD2O1

97. Zander C. Denmark reports first case of Zika virus. Patient, currently hospitalized, had been traveling in Latin America. 2016 Jan 27 [cited 2016 Feb 10]. http://www.wsj.com/articles/denmark-reports-first-case-of-zika-virus-1453895642

98. Baronti C, Piorkowski G, Charrel RN, Boubis L, Leparc-Goffart I, de Lamballerie X. Complete coding sequence of Zika virus from a French Polynesia outbreak in 2013. Genome Announc. 2014;2:e00500–14. http://dx.doi.org/10.1128/genomeA.00500-14

99. Tappe D, Rissland J, Gabriel M, Emmerich P, Gunther S, Held G, et al. First case of laboratory-confirmed Zika virus infection imported into Europe, November 2013. Euro Surveill. 2014;19:20685. http://dx.doi.org/10.2807/1560-7917.ES2014.19.4.20685

100. World Health Organization. Zika virus infection—Haiti. 2016 Jan 21 [cited 2016 Feb 3]. http://www.who.int/csr/don/21-january-2016-zika-haiti/en/

101. Zammarchi L, Tappe D, Fortuna C, Remoli ME, Günther S, Venturi G, et al. Zika virus infection in a traveller returning to Europe from Brazil, March 2015. Euro Surveill. 2015;20:21153. http://dx.doi.org/10.2807/1560-7917.ES2015.20.23.21153

102. European Centre for Disease Prevention and Control. Epidemiological update: outbreaks of Zika virus and complications potentially linked to the Zika virus infection. 2015 Dec 18 [cited 2016 Feb 3]. http://ecdc.europa.eu/en/press/news/_layouts/forms/News_DispForm.aspx?ID=1342&List=8db7286c-fe2d-476c-9133-18ff4cb1b568&Source=http%3A%2F%2Fecdc%2Eeuropa%2Eeu%2Fen%2Fpress%2Fepidemiological_updates%2FPages%2Fepidemiological_updates%2Easpx

103. Rijksinstituut voor Volksgezondheid en Milieu. Zika virus also in Curaçao. 2016 Jan 28 [cited 2016 Feb 8]. http://www.rivm.nl/Documenten_en_publicaties/Algemeen_Actueel/Nieuwsberichten/2016/Zikavirus_ook_op_Cura%C3%A7ao

104. Decavale J. Portugal has five mild cases of Zika virus, all after Brazil trips. Reuters. 2016 Jan 27 [cited 2016 Feb 8]. http://www.reuters.com/article/health-zika-portugal-idUSKCN0V5256

105. De Sanidad M, Sociales e Igualdad S. Weekly update of Zika cases confirmed by the National Center for Microbiology. 2016 Feb 4 [cited 2016 Feb 8]. http://www.msssi.gob.es/gabinete/notasPrensa.do?id = 3895

106. Huuhtanen M. Danish hospital says case of Zika virus discovered. Associated Press. 2016 Jan 27 [cited 2016 Feb 8]. http://bigstory.ap.org/article/4491870e5b274776bf91780a19457b58/danish-hospital-says-case-zika-virus-discovered

107. Public Health England. Zika virus. Clinical advice on Zika: assessing pregnant women following travel; symptoms, transmission (includes sexual transmission), epidemiology. GOV.UK. 2016 Feb 17 [cited 2016 Feb 17]. https://www.gov.uk/guidance/zika-virus

108. Kwong JC, Druce JD, Leder K. Zika virus infection acquired during brief travel to Indonesia. Am J Trop Med Hyg. 2013;89:516–7. http://dx.doi.org/10.4269/ajtmh.13-0029

109. Pyke AT, Daly MT, Cameron JN, Moore PR, Taylor CT, Hewitson GR, et al. Imported Zika virus infection from the Cook Islands into Australia, 2014. PLoS Curr. 2014;6:6.

110. Schwan K. Zika virus—Pacific (07): Chile (Easter Island), French Polynesia. ProMed. 2014 Mar 3 [cited 2016 Feb 10]. http://www.promedmail.org/post/2322907, archive no. 20140309.2322907.

111. Cooperativa (Chile). Zika virus—Pacific (15): Chile (Easter Island) ex Tahiti. ProMed. 2014 Sept 25 [cited 2016 Feb 10]. http://www.promedmail.org/post/2804821, archive no. 20140925.2804821.

112. World Health Organization Western Pacific Region. Pacific syndromic surveillance report—Week 33, ending 16 August, 2015. 2015 Aug 16 [cited 2016 Feb 3]. http://www.wpro.who.int/southpacific/programmes/communicable_diseases/disease_surveillance_response/PSS-16-August-2015/en/

113. New Zealand Ministry of Health. Zika virus update. The Ministry of Health has extended its Pacific travel advice around the Zika virus to include Tonga as well as Samoa as an area of active transmission. 2016 Jan 29 [cited 2016 Feb 3]. http://www.health.govt.nz/news-media/media-releases/zika-virus-update

114. Centers for Disease Control and Prevention. Zika virus in the Pacific Islands. 2016 Feb 12 [cited 2016 Feb 16]. http://wwwnc.cdc.gov/travel/notices/alert/zika-virus-south-pacific

115. World Health Organization Western Pacific Region. Pacific syndromic surveillance report, Week 17, ending 26 April, 2015. 2015 Apr 26 [cited 2016 Jan 29]. http://www.wpro.who.int/southpacific/programmes/communicable_diseases/disease_surveillance_response/PSS-26-April-2015/en/

116. World Health Organization. Zika virus infection—Guyana, Barbados and Ecuador. 2016 Jan 20 [cited 2016 Feb 8]. http://www.who.int/csr/don/20-january-2016-zika-guyana-barbados-ecuador/en/

117. World Health Organization. Zika virus infection—Bolivia. 2016 Jan 20 [cited 2016 Feb 8]. http://www.who.int/csr/don/20-january-2016-zika-bolivia/en/

118. Herriman R. Zika virus—Brazil (05). ProMed. 2015 Jun 12 [cited 2016 Feb 8]. http://www.promedmail.org/post/3431148, archive no. 20150612.3431148.

119. Castelo Branco B. Zika virus—Brazil (06): (Bahia). ProMed. 2015 Jun 19 [cited 2016 Feb 8]. http://www.promedmail.org/post/3449500, archive no. 20150619.3449500.

120. Zika virus—Brazil. (07). ProMed. 2015 Jun 30 [cited 2016 Feb 8]. http://www.promedmail.org/post/3473420, archive no. 20150630.3473420.

121. Zika virus—Brazil. (08). ProMed. 2015 July 16 [cited 2016 Feb 8]. http://www.promedmail.org/post/3513770, archive no. 20150716.3513770.

122. Associated Press. The latest: Chile reports its first 3 confirmed Zika cases. 2016 Feb 2 [cited 2016 Feb 8]. http://bigstory.ap.org/article/52f57f3f5e0f48f5a9469acad6318faf/latest-4th-case-zika-identified-spain

123. Instituto Nacional de Salud (National Institutes of Health Colombia). Epidemiologic week number 3 2016 (17 Jan–23 Jan). 2016 Jan 23 [cited 2016 Feb 8]. http://www.ins.gov.co/boletin-epidemiologico/Boletn%20Epidemiolgico/2016%20Boletin%20epidemiologico%20semana%203.pdf

124. World Health Organization. Zika virus infection—Guatemala. 2015 Nov 27 [cited 2016 Feb 8]. http://www.who.int/csr/don/27-november-2015-zika-guatemala/en/

125. World Health Organization. Guillain-Barré syndrome—El Salvador. 2016 Jan 21 [cited 2016 Feb 8]. http://www.who.int/csr/don/21-january-2016-gbs-el-salvador/en/

126. World Health Organization. Zika virus infection—Honduras. 2015 Dec 21 [cited 2016 Feb 8]. http://www.who.int/csr/don/21-december-2015-zika-honduras/en/

127. World Health Organization. Zika virus infection—Panama. 2015 Dec 22 [cited 2016 Feb 3]. http://www.who.int/csr/don/22-december-2015-zika-panama/en/

128. World Health Organization. Zika virus infection—Paraguay. 2015 Dec 3 [cited 2016 Feb 3]. http://www.who.int/csr/don/03-december-2015-zika-paraguay/en/

129. World Health Organization. Zika virus infection—France–Saint Martin and Guadeloupe. 2016 Jan 21 [cited 2016 Feb 3]. http://www.who.int/csr/don/21-january-2016-zika-france/en/

130. Enfissi A, Codrington J, Roosblad J, Kazanji M, Rousset D. Zika virus genome from the Americas. Lancet. 2016;387:227–8. http://dx.doi.org/10.1016/S0140-6736(16)00003-9

131. World Health Organization. Zika virus infection—Venezuela. 2015 Dec 3 [cited 2016 Feb 3]. http://www.who.int/csr/don/03-december-2015-zika-venezuela/en/
